# Original and generic preservation solutions in organ transplantation. A new paradigm? ^1^

**DOI:** 10.1590/s0102-865020200010000001

**Published:** 2020-03-09

**Authors:** Joan Roselló-Catafau, Arnau Panisello-Roselló, Gianfranco Pasut, Miquel Navasa, Jacques Pirenne, René Adam

**Affiliations:** I PhD, Experimental Pathology Department, Institute of Biomedical Research (IIBB-CSIC), Catalonia, Barcelona, Spain. Manuscript writing, final approval.; II PhD, Department of Pharmaceutical and Pharmacological Sciences, Padova, Italy. Manuscript writing, final approval.; III PhD, Liver Transplantation Unit, Hospital Clínic, Barcelona, Catalonia, Spain. Manuscript writing, final approval.; IV PhD, Abdominal Transplant Surgery UZ Leuven, University Hospitals Leuven, Belgium. Manuscript writing, final approval.; V PhD, Centre Hépatobiliare, AP-HP Hôpital Paul Brousse, Inserm U935, Université Paris-Sud, Villejuif, France. Manuscript writing, final approval.

**Keywords:** Organ Preservation Solutions, Polyethylene Glycols, Glutathione, Calcium

## Abstract

Solid organ transplantation is a very complex process, in which the storage of the graft in a preservation solution is mandatory in order to extend ischemic times and contain further damage. The condition in which the organ is transplanted is critical for the outcome of the organ recipient. The recent emergence of generic versions of organ preservation solutions (solutions with the same composition and under the same legislation as the original versions, but with different brands) compelled us to study whether the standards are maintained when comparing the original and its generic counterpart. Along these lines, we discuss and comment on some aspects concerning this issue of general interest in the organ transplantation field.

## Introduction

The use of generic preservation solutions (GPS) is increasing and, as has happened in other fields such as in the case of immunosuppressants, their efficacies vary. With this in mind, it seems reasonable that the concerns about the efficacy of GPS should be addressed in the context of regulatory requirements for quality and for composition in clinical studies to guarantee the best clinical practice in organ transplantation

The recent use of generic organ preservation solutions for clinical transplantation has motivated us to determine whether their efficacy is the same as that of the original versions. This controversial issue has been raised by several recent studies, which have questioned the bioequivalence of some generic immunosuppressants compared with their original counterpart^1 - 3^ .

The recent emergence of GPS provides more options to physicians for choosing the most suitable one for organ transplantation. However, although the composition might be the same, other factors (i.e., purity of the components of the solution, performance/quality of the bags and storage conditions) could affect the initial composition of the solution and, consequently, graft viability and clinical outcome in the organ recipient. In line with this, it has been recently demonstrated that the type of preservation solution affects graft function and outcome, independently of other factors^4 - 7^ .

By definition, “generics” are created to have the same composition, safety, quality, and performance characteristics as that of an existing approved original product. It is surprising to discover that different solutions defined as “generics” have been used for several years in Europe, although they have different quality requirements and different classifications according to regulatory CE rules (such as Drug, MD Class III or MD Class IIa). In this sense, it seems important to point out that some CE-marked generics containing labile molecules in their formulations (such as glutathione and polyethylene glycols)^8^ are claimed to have longer expiration periods at room temperature (up to 2 years according to the suppliers) compared to their corresponding original preservation solutions (OPS) that are stored at 4°C^9 , 10^ . This indicates differences in composition of the OPS and GPS used in organ transplantation, which is a matter of controversy as discussed below.

The quality of preservation is a key factor during organ transplantation. There is an increasing tendency to resort to preservation solutions, because of the lack of optimal organs (i.e., non-steatotic grafts)^10^ . Therefore, optimal conditions for preservation solutions are mandatory to increase the available donor pool, especially for marginal grafts such as steatotic liver grafts, thereby allowing donations from donors after cardiac death that usually have a higher risk of primary non-function or dysfunction after transplantation^13^ , since they are more vulnerable to ischemia-reperfusion injury (IRI)^11 , 12^ . This is a concerning matter, as preservation solutions can contain components such as polyethylene glycols (PEGs) and glutathione^14 - 16^ , which are very sensitive to the external environment conditions that can promote their degradation.

PEGs are non-toxic polymers that are partly responsible for protecting the organ against IRI^17 - 19^ . PEG is known to contribute to the activation of endothelial nitric oxide synthase (involved in nitric oxide generation) and other cytoprotective agents such as AMP-activated protein kinase^19^ . In this sense, PEG is a key factor and anything that affects the integrity of this molecule will have an impact on the performance of the solution. Light has a harmful effect on PEGs and long exposures to ultraviolet radiation can damage the polymeric backbone of PEG^20^ , which is enhanced by increased temperatures^21^ . PEGs can be degraded by the formation of peroxides, which cleave the polymeric backbone and consequently lead to the loss of oncotic properties. The formation of peroxides is triggered by the presence of oxygen and is catalyzed by light, metals, and an increase in temperature. In general, a higher temperature of storage means greater degradation^21^ and although the composition is the same, the preservation conditions can harm the initial properties of the compounds in the solution.

Glutathione is present in its reduced form and is another critical component often used in preservation solutions^22^ . Its antioxidant properties are essential for organ preservation, especially in preparing the graft during reperfusion. Reduced glutathione is a very labile component and its auto-oxidation is accelerated by an increase in temperature and contact with oxygen. Long periods of storage at room temperature are not recommended for storing any preservation solutions containing glutathione^23 , 24^ .

**Figure 1:**
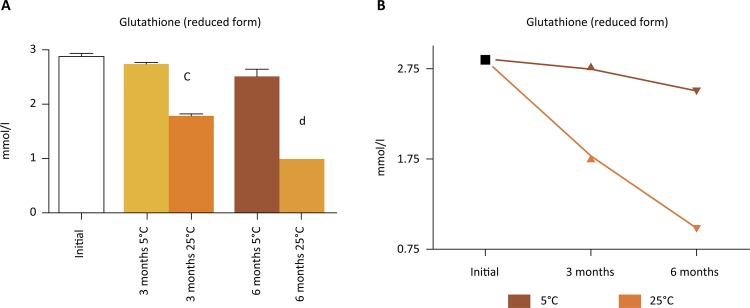
Reduced glutathione levels (mmol/l) in preservation solutions from the same batch stored at different temperatures (5°C and 25°C) and evaluated at different times (initial, three months and six months). Results are expressed as mean ± SEM ( *n* = 3). a. *p* < 0.0005 indicates significant differences when comparing to 3 months at 5°C. b. *p* < 0.0005 indicates significant differences when comparing to 6 months at 5°C. Figure 1 *b* shows the degradation profile of reduced glutathione in the IGL-1 batches stored at 5°C or 25°C.

Finally, we investigated the impact of temperature on the glutathione content of different IGL-1 batches stored for 6 months at 25°C and 5°C respectively. Analyses confirmed a significant decrease in reduced glutathione levels in the IGL-1 solution kept at room temperature for six months (30%) compared to the one stored at 5°C (according to the supplier’s instructions). Data shown in Figure 1 revealed that room temperature affected glutathione degradation in IGL-1 solutions, confirming that IGL-1 batches stored at room temperature are not suitable for use in transplantation.

We evaluated the composition of two preservation solutions, one corresponding to the original (Celsior) and the other one to its European generic counterpart. Our results revealed decreased levels of reduced glutathione (0.51 mg/ml *vs* . 0.89 mg/ml) and increased levels of calcium (0.312 mmol/l *vs* . 0.258 mmol/l) in the generic solution compared to the original Celsior solution, with both components being out of range in the generic solution. Moreover, the higher calcium concentration in the generic solution could have a harmful deleterious effect on abdominal organ preservation^25 , 26^ . The generic solution also had a significantly increased lack of transparency (0.13 *vs.* 0.00) when measuring absorbance at 340 nm. These results by themselves, obtained and validated by an external laboratory (Catalent Pharma Solutions, Limoges, France) following European Pharmacopoeia Methods, would justify reasonable doubt about the quality of the generic solution, as well as about other factors such as the quality of the product (purity of the components in the solutions) and manufacturing procedures (i.e., bag quality/performance). Therefore, it seems reasonable that these concerns about the efficacy of generic preservation solutions should be addressed in the context of regulatory requirements for quality, composition testing and clinical studies.

Finally, it is important to point out that the reduced expenses of using GPS would not compensate for the additional costs resulting from a potentially worse transplant outcome.

## Conclusions

We address several concerns regarding the use of generic preservation solutions, which could affect the quality of graft preservation and transplant outcomes. Our common goal is to guarantee the best clinical practice in organ transplantation to ensure good clinical outcome in organ recipients.
